# Trends in the Burden of Vision Impairment in Saudi Arabia, 1990–2023: A Global Burden of Disease Study Analysis

**DOI:** 10.3390/jcm15197682

**Published:** 2026-10-04

**Authors:** Ahmed Alduais, Abdullah Alduais

**Affiliations:** 1 Department of Psychology, Norwegian University of Science and Technology, NO-7491 Trondheim, Norway; 2 Disability and Access Services Unit, English Language Institute, King Abdulaziz University, Jeddah 21589, Saudi Arabia; aalduais@kau.edu.sa

**Keywords:** Global Burden of Disease, vision impairment, Saudi Arabia, diabetic retinopathy, epidemiological trend, epidemiologic transition

## Abstract

**Background:** Vision impairment (VI) in Saudi Arabia is common, largely preventable or treatable, and increasingly linked to chronic disease rather than solely to the causes that have historically dominated blindness. Recent Saudi-specific evidence places adult VI prevalence at approximately 17% and blindness at under 2%, with diabetic retinopathy affecting roughly one in three Saudi adults with diabetes; however, no study has yet examined how this burden has trended nationally over time using the full 1990–2023 window made available by the Global Burden of Disease (GBD) 2023 round. This study quantified trends in the burden of VI in Saudi Arabia by severity, cause, sex, and age, and characterised the contribution of population ageing to the observed pattern. **Methods:** This repeated cross-sectional trend analysis used GBD 2023 national-level modelled estimates for Saudi Arabia (1990–2023). The estimated annual percentage change (EAPC) was calculated via log-linear regression on age-standardised prevalence and years-lived-with-disability (YLD) rates. A Durbin-Watson check confirmed autocorrelation in every annual series, and because GBD point estimates carry their own reported uncertainty, EAPC confidence intervals were derived two further ways alongside the naive ordinary-least-squares (OLS) interval: a Newey-West heteroskedasticity- and autocorrelation-consistent (HAC) interval, and a Monte Carlo interval that resampled each annual estimate within its GBD-reported 95% uncertainty interval. A trend is reported here as confirmed only where all three methods agreed on direction, and as suggestive where they did not. Segmented-regression sensitivity checks, cause decomposition, and comparison of age-standardised against crude rates were used to characterise breakpoints and the contribution of ageing. **Results:** Age-standardised blindness and severe VI declined substantially and robustly (blindness prevalence EAPC = −2.60%/year, confirmed decreasing in females and males separately, both *p* < 0.05 on a formal sex-difference test), while moderate VI rose robustly overall and among males specifically (EAPC = +0.61%/year); the female-specific rise in moderate VI, and the decline in presbyopia, were directionally consistent but not robust to the choice of uncertainty method. Diabetes-attributable rates increased faster than any other tracked cause and did so robustly under every uncertainty specification (crude prevalence EAPC ≈ +4.47%/year), and males carried a disproportionate, formally sex-different share of diabetes-attributable blindness relative to females; these tracked causes covered only 44–51% of the total VI envelope, so cause-specific conclusions are restricted to the causes examined. The largest proportional increases in case counts were concentrated in working-age adults (30–59 years) rather than older adults, and the gap between crude and age-standardised trends is consistent with an important contribution from Saudi Arabia’s changing age structure, although the study design does not support assigning a specific numerical fraction to that contribution. Segmented-regression analysis further indicated that the national decline in blindness and the rise in moderate VI were concentrated in 1990–2019 and 1990–2008 respectively, with the post-breakpoint segments no longer showing a statistically distinguishable trend. **Conclusions:** Saudi Arabia’s VI burden is shifting in composition rather than simply declining. Diabetes-related causes, sex differences, and working-age adults emerge as priorities for vision-health policy, and the post-breakpoint plateaus identified here warrant continued monitoring in future GBD rounds.

## 1. Introduction

Vision impairment (VI) in Saudi Arabia is common, largely preventable or treatable, and already a present-day public health concern rather than a distant projection. A 2025 systematic review and meta-analysis pooling nine Saudi studies (N = 29,707 adults) estimated adult VI prevalence at 16.8% (95% CI [11.4, 23.9]) and blindness at 1.7%. Uncorrected refractive error was the leading cause of impairment and cataract the leading cause of blindness, with prevalence estimates rising across the pooled studies [1]. Diabetic retinopathy, a chronic-disease-driven cause of VI rather than one of the historically dominant avoidable causes, affects an estimated 31% of Saudi adults with diabetes overall and 24% of those with type 2 diabetes specifically, with poor glycaemic control and longer diabetes duration as the strongest predictors [2]. Saudi Arabia’s adult diabetes prevalence itself is estimated at 21–32% and rising [3,4], placing a large and growing population at risk of diabetes-attributable eye disease. Screening uptake, meanwhile, remains low: a 2025 primary-care survey in Riyadh found only 9.1% of Saudi attendees had ever received a vision screening, with uptake associated more with chronic-disease status than with demographic factors alone [5]. Together, these figures establish that the problem is substantial (roughly one in six Saudi adults affected), current (estimates drawn from studies conducted within the past decade), shifting in cause (a growing diabetes-attributable share), and inadequately addressed at the health-system level (screening uptake below one in ten).

This pattern is consistent with the epidemiologic transition long described for populations undergoing demographic and economic change: as mortality falls, life expectancy rises, and health systems mature, the dominant disease profile shifts from acute and infectious causes toward chronic and degenerative ones [6]. Saudi Arabia’s own historical VI literature traces exactly this shift. Early population-based surveys conducted in the 1990s described a burden of blindness and VI still substantially attributable to avoidable causes: in Bisha, cataract accounted for 52.6% of blindness and refractive error for 67.9% of VI, while trachoma, though present, was already a minor contributor [7,8]. Over the following two decades, successive cross-sectional surveys documented a clear shift in cause structure. Studies conducted in northern Saudi Arabia and Arar in the 2000s and 2010s attributed a growing share of VI to refractive error, cataract, diabetic retinopathy, and glaucoma [9,10], while a rapid assessment in Taif linked posterior segment disease and diabetes-related retinopathy to a rising share of avoidable blindness [11]. More recent, national-level evidence extends this pattern to the present: a 2025 systematic review of the Qassim region and a broader Arab-region meta-analysis both continue to implicate diabetic retinopathy and glaucoma alongside cataract as leading contributors to current Saudi and regional VI burden (see Section 5). Mokdad et al. [12] situate this same shift within the broader epidemiological transition documented across the Arab world, where non-communicable and chronic conditions have progressively displaced infectious and childhood causes of disease burden—the regional backdrop against which the present study’s national-level test of that transition, described next, is set.

Two structural mechanisms account for this shift at the regional and global level: population ageing and rising diabetes prevalence. GBD-based analyses attribute the growing burden of blindness and VI to population ageing, since cataract, glaucoma, and age-related macular degeneration disproportionately affect older adults, and age-standardisation is the standard method used to isolate this ageing effect from genuine changes in disease rates [13,14,15]. In parallel, the rising prevalence of type 2 diabetes across the Gulf region has been linked in GBD-based studies to an increasing burden of diabetic retinopathy and diabetes-attributable vision loss, with projections suggesting continued growth in countries where diabetes prevalence continues to rise [16,17,18,19]. Saudi Arabia, with one of the highest reported prevalences of diabetes in the Middle East and North Africa (MENA) region, sits at the intersection of both drivers.

Even so, no study has yet produced a Saudi Arabia-specific, GBD-based trend analysis of VI burden spanning the full 1990–2023 period made available in the GBD 2023 round. The closest available evidence is regional rather than national. A MENA-wide GBD analysis found that age-standardised prevalence and years-lived-with-disability rates for vision loss fell between 1990 and 2019 even as absolute case numbers rose, with the largest prevalent burden concentrated in the 65–69 age group [20]. That analysis pools Saudi Arabia with 20 other countries of markedly different socio-demographic profiles, stops at 2019, and does not report country-specific estimated annual percentage change (EAPC), cause-specific decomposition, or severity-level trends for Saudi Arabia. What remains of the Saudi-specific evidence base is a set of geographically fragmented, methodologically heterogeneous cross-sectional surveys [21]. These surveys cannot, by design, quantify a national trend, and none can statistically test whether observed changes in burden are attributable to population ageing, population growth, or genuine shifts in age-specific disease rates.

## 2. Problem Statement

The problem this study addresses is not simply that a particular statistical analysis has not yet been performed. Saudi Arabia is undergoing rapid demographic and epidemiological transition: its population is ageing, its diabetes prevalence is among the highest in the MENA region, and its documented cause structure for VI has shifted, within the historical record summarised above, from predominantly avoidable causes toward a mix increasingly influenced by chronic disease. At the same time, the available Saudi-specific VI evidence is fragmented across small, geographically localised, methodologically heterogeneous surveys conducted at different times with different instruments [21,22,23], and the one recent attempt to synthesise this evidence quantitatively [1] is a pooled cross-sectional meta-analysis, not a national trend analysis; it can report a current prevalence estimate but not how that burden has moved over time, by how much, for whom, or why.

This absence of a national trend picture has practical consequences. Without knowing whether the burden of blindness, severe VI, moderate VI, and presbyopia is moving in the same direction or different directions, health authorities cannot tell whether historically successful investments, such as cataract surgical coverage, remain the correct priority or whether resources should shift toward a different and growing problem. Without a cause-specific national trend, policymakers cannot judge whether diabetic retinopathy screening and diabetes prevention deserve a larger share of vision-health investment relative to established blindness-prevention programmes. Without an age-specific national trend, service planners cannot judge whether working-age adults, rather than only older adults, should be a target population for screening and early intervention. In short, health systems may continue to prioritise the historical causes of blindness while underrecognising an emerging, chronic-disease-driven burden of moderate VI, and they currently have no Saudi-specific national evidence base with which to make that judgement. This is the problem the present study is designed to address.

## 3. Purpose of the Inquiry

The purpose of this repeated cross-sectional trend analysis is to examine changes in the burden of vision impairment in Saudi Arabia from 1990 through 2023 using Global Burden of Disease (GBD) 2023 national-level modelled estimates, and to characterise how that burden differs by severity, cause, sex, and age. The analysis is conducted now because the GBD 2023 round is the first to make a full, internally consistent 1990–2023 series available for Saudi Arabia; but the scientific and policy rationale for extending the analysis to 2023 is not simply that the data exist. A four-year extension beyond the most recent prior regional benchmark (to 2019) [20] captures the period in which Saudi Arabia’s diabetes epidemic and demographic ageing have continued to progress, and, as the segmented-regression results below show, it is also the period in which several severity-specific trends appear to have changed pace. Extending the observation window is therefore expected to contribute new knowledge about whether the divergence between falling blindness and rising moderate VI, documented regionally through 2019, has continued, accelerated, or begun to plateau nationally in Saudi Arabia specifically.

The study has three aims: (1) to quantify the estimated annual percentage change in the prevalence and years-lived-with-disability rate of VI in Saudi Arabia between 1990 and 2023, overall, by severity category, and by sex, testing formally whether male and female trends differ; (2) to determine which causes have contributed most to the changing burden among the causes examined and how their relative contribution and rate of change have shifted over the study period; and (3) to characterise the age distribution of the burden and assess whether the divergence between crude and age-standardised trends is consistent with an important contribution from population ageing, as opposed to genuine changes in age-specific rates.

## 4. Originality, Rationale, and Significance of the Inquiry

### 4.1. Originality

This study is, to our knowledge, the first Saudi Arabia-specific GBD-based trend analysis of VI burden. It differs from the existing Saudi survey literature by using a single, internally consistent estimation framework applied uniformly across 34 years, and it differs from the existing MENA-regional GBD analyses [20,24] by isolating Saudi Arabia rather than pooling it with up to 20 countries of markedly different socio-demographic and diabetes-prevalence profiles, by extending the observation window four years beyond the most recent regional benchmark, and by decomposing the burden simultaneously by severity category, cause, sex, and age within a single national dataset.

### 4.2. Rationale

A repeated cross-sectional, GBD-based national trend design is the logically necessary approach given the state of the Saudi evidence base described in Section 2: no single Saudi survey or administrative dataset covers the full 1990–2023 period with consistent methodology, so a synthesised, modelled series is the only source from which a national trend can currently be constructed; the study’s questions concern population-level change, for which aggregate annual estimates suffice; and a repeated cross-sectional design is what a fragmented, non-cohort evidence base of this kind calls for, in contrast to a single-point prevalence estimate that cannot distinguish a genuinely changing disease profile from a stable one.

### 4.3. Significance

If Saudi Arabia’s VI burden is shifting in composition as the epidemiologic-transition literature would predict, and as the historical Saudi survey record already suggests, then national trend evidence has direct value for research (establishing a national baseline against which future GBD rounds and future Saudi surveys can be compared), clinical practice (informing which conditions ophthalmology and endocrinology services should anticipate seeing more of), and health-system planning and policy (informing whether diabetic retinopathy screening, refractive services, and low-vision rehabilitation warrant a larger share of vision-health investment alongside established blindness-prevention programmes). These are the contributions this study is positioned to make; the extent to which the data in fact support them is addressed empirically in Section 7 and weighed against the study’s methodological limitations in Section 8.

## 5. Literature Review

### 5.1. Global and Regional Trends in Blindness and Vision Impairment

The most influential single source for this study’s design is Steinmetz et al. [25], the GBD 2020 global analysis of causes and trends in blindness and VI over 30 years. It establishes the pattern this study tests nationally: age-standardised blindness prevalence has fallen globally even as the absolute number of people living with VI has risen, because population growth and ageing outpace the rate of improvement. It also establishes the design language, “repeated cross-sectional trend analysis,” and the EAPC-based analytic approach that the present study adopts. Regionally, Bahremani et al. [20] and Qaddoumi et al. [24] apply the same GBD framework to the Middle East and North Africa, both reporting the same divergent pattern, falling blindness alongside a growing overall vision-loss burden, but neither is able to isolate Saudi Arabia from the 20 or more pooled countries or to extend past 2019. These two studies define the regional benchmark against which the present, Saudi-specific analysis is compared throughout Section 8.

### 5.2. Saudi-Specific Prevalence and Causes

Al Khathami et al. [1], a 2025 systematic review and meta-analysis pooling nine Saudi studies, is the most directly relevant Saudi-specific source: it provides the current pooled prevalence estimates (VI 16.8%, blindness 1.7%) used in Section 1 and confirms that uncorrected refractive error and cataract remain the leading causes of impairment and blindness respectively, while noting that prevalence estimates have risen across the studies it pools. This is a cross-sectional synthesis, not a trend analysis, so it establishes current magnitude but not the direction or pace of change that the present study is designed to supply. The historical Saudi survey literature reviewed in Section 1 [7,9,10,11,22,23] contributes the record of a shifting cause structure over time that this study’s cause-decomposition results are read against.

### 5.3. Diabetic Retinopathy and Diabetes-Attributable Vision Loss

Alshahrani et al. [2], a systematic review and meta-analysis of 11 Saudi studies, is the second key empirical source: it establishes that diabetic retinopathy affects approximately 31% of Saudi adults with diabetes overall (24% among those with type 2 diabetes specifically), with glycaemic control and diabetes duration as the strongest predictors. Globally, Meng et al. [17] report that diabetic retinopathy is an increasingly important driver of VI specifically among the working-age population, a finding against which this study’s age-period results are interpreted in Section 8, and Cao et al. [16] project continued growth in diabetes-attributable blindness and vision loss globally to 2050. Together, these sources motivate treating diabetes-attributable VI as a distinct analytic focus (Aim 2) rather than folding it into an undifferentiated cause list.

### 5.4. Demographic Ageing and Burden Change

Yin et al. [13] is the key source for interpreting the comparison between age-standardised and crude EAPCs used to characterise the role of ageing (Aim 3): it demonstrates, in a global GBD analysis, that population ageing is a major structural driver of rising absolute vision-loss burden even where age-specific risk is falling, which is the same logic this study applies to the Saudi-specific comparison.

### 5.5. Sex Differences and Age-Specific Patterns

No Saudi-specific GBD source formally tests sex differences in VI trend, which is part of the gap this study addresses (Aim 1); the present analysis therefore draws on national diabetes-prevalence sex-trend data [26] to interpret any sex differences found. Age-specific patterns are interpreted against the same working-age-diabetes literature cited above [17].

### 5.6. Access to Screening and Ophthalmic Services

Elmetwally et al. [5], a 2025 cross-sectional survey of Saudi primary-care attendees in Riyadh, found vision-screening uptake of only 9.1%, associated more strongly with chronic-disease status than with demographic characteristics. This is the most direct Saudi-specific evidence on the health-services-access side of the discussion in Section 8 and is read alongside the Andersen Behavioral Model below.

### 5.7. Theoretical Frameworks

Two conceptual frameworks organise the interpretation offered in Section 8; both are used, consistent with their appropriate role in an ecological, GBD-based trend analysis, to frame plausible explanations for the observed population-level patterns rather than as constructs measured directly in this study.

Epidemiologic Transition Theory [6] proposes that as populations undergo mortality decline, rising life expectancy, and social and health-system change, the dominant pattern of disease burden shifts from acute and infectious causes toward chronic and degenerative ones. This framework is directly relevant here because the historical Saudi VI literature already documents a cause-structure shift consistent with this prediction (from trachoma and cataract-dominated blindness toward a mix increasingly influenced by diabetic retinopathy and glaucoma), and because the present study’s central empirical question, whether moderate VI and diabetes-attributable VI are rising while historically dominant causes decline, is precisely the pattern the theory predicts for a population at Saudi Arabia’s stage of transition.

The Andersen Behavioral Model of Health Services Use [27] proposes that health-service utilisation is shaped by predisposing characteristics (e.g., age, sex, health beliefs), enabling resources (e.g., income, service availability, health-system capacity), and perceived or evaluated need. This model is used in Section 8 to frame, as hypotheses for future research rather than as tested mechanisms, why diabetic-retinopathy screening uptake in Saudi Arabia is low [5] and why the sex differences reported in Section 7 might exist; the model’s predisposing, enabling, and need constructs were not measured in the present GBD-based analysis, and no claim is made that they were.

## 6. Methods

### 6.1. Design

This study is a repeated cross-sectional trend analysis of Saudi Arabia’s VI burden using estimates from the Global Burden of Disease Study 2023 (GBD 2023), covering the period 1990–2023. GBD estimates are independent, population-level modelled estimates produced for each calendar year rather than repeated measurements on the same individuals, so the term longitudinal is avoided throughout, consistent with the design language used in prior GBD-based vision-loss trend studies [25,28].

A repeated cross-sectional trend design is the appropriate choice for three reasons specific to this research question. First, no single Saudi survey or administrative dataset covers the full 1990–2023 period with consistent methodology; GBD instead synthesises the fragmented primary literature described in Section 1 into internally consistent, comparable annual estimates using a standardised modelling framework applied uniformly across countries and years [29,30]. A repeated cross-sectional design is what that kind of synthesised, year-by-year estimate calls for, since there is no cohort to follow. Second, the study’s central questions are about population-level change over time rather than about individual risk trajectories, so aggregate annual estimates are sufficient to answer them and do not require individual-level data. Third, Saudi Arabia is explicitly undergoing the kind of demographic and epidemiological transition for which trend analysis, rather than a single cross-sectional prevalence estimate, is the informative unit of analysis [6,12]; a snapshot at one time point cannot distinguish a genuinely changing disease profile from a stable one, which is precisely the distinction this study needs to draw.

### 6.2. Data Source and Analytic Dataset

This study analysed aggregate, national-level modelled estimates produced by the GBD 2023 round; no individual-level records were obtained or analysed, and the study therefore has no sample of participants in the conventional epidemiological sense. The analytic dataset comprised GBD’s modelled estimates of the burden of blindness and vision loss in the Saudi Arabian population, all ages, for both sexes combined and disaggregated by sex, for each year from 1990 to 2023 (34 annual estimates per series). Data were obtained from the GBD Results Tool (GBD 2023 release; Institute for Health Metrics and Evaluation, IHME) [31], restricted to location = Saudi Arabia.

Variables were selected and named following the GBD Results Tool’s own filter structure, so that the extracted files remain traceable back to the specific query that produced them. Every GBD extract is defined by six filterable dimensions: measure (the health outcome, e.g., Prevalence or YLDs), location (fixed to Saudi Arabia here), sex (Male, Female, or Both), age (either a specific age band, an aggregate such as “All ages,” or “Age-standardized”), cause (the underlying disease, e.g., Cataract), and rei (short for “risk, exposure, or impairment” in GBD terminology—here used for the severity category, e.g., Blindness), together with the metric in which results are expressed (Number, Percent, or Rate) and year. The full filter specification for each of the six extracts used in this study, including exact GBD Results Tool selections and output filenames, is available in the OSF repository (see Data Availability Statement) rather than reproduced in the main text, to keep this section focused on the analytic dataset itself; it remains fully reproducible from the header comments of the replication script (01_analysis.R) and the openly deposited raw files.

### 6.3. Measures

GBD case definitions. GBD classifies vision loss by presenting visual acuity in the better eye, following World Health Organization International Classification of Diseases thresholds: blindness is presenting visual acuity worse than 3/60; severe vision impairment is presenting visual acuity from 3/60 up to (but not including) 6/60; moderate vision impairment is presenting visual acuity from 6/60 up to (but not including) 6/18; and presbyopia is an age-related loss of near-vision accommodation, defined separately from the distance-acuity-based categories above and captured by GBD as an uncorrected-near-vision impairment. These four categories are mutually exclusive within GBD’s “Blindness and vision loss” impairment hierarchy. Years lived with disability (YLDs) are calculated by GBD as the product of a condition’s prevalence and a disability weight, a value between 0 (full health) and 1 (a state judged, via population survey methods, equivalent to death) that reflects the severity of functional loss associated with that health state; because disability weights rise sharply with severity, a given number of prevalent blindness cases contributes disproportionately more YLDs than the same number of moderate VI cases, which is why prevalence and YLD results can diverge for the same cause or severity category, as they do for diabetes-attributable VI in Section 7.2.

Three GBD outcome measures were used: Prevalence, Years Lived with Disability (YLDs), and the derived metrics Number, Percent, and Rate (crude and age-standardised). Burden was examined at three levels of granularity: an aggregate impairment envelope (“Blindness and vision loss”); four mutually exclusive severity categories (blindness, severe VI, moderate VI, and presbyopia); and nine attributable causes initially extracted (cataract, glaucoma, refractive disorders, age-related macular degeneration, diabetes mellitus [combined, type 1, and type 2], diabetes and kidney diseases, and the aggregate cause envelope). For clinical clarity, “refraction disorders” is reported here as uncorrected refractive error, and, where the manuscript discusses diabetes as a cause of VI, the underlying ocular pathology, diabetic retinopathy (and, for macular involvement, diabetic macular oedema), is named explicitly. GBD’s own cause label in the downloaded extracts is “Diabetes mellitus,” reflecting that GBD attributes vision loss to the parent metabolic disease rather than coding diabetic retinopathy as a separate cause label, and this correspondence is stated explicitly here so that “diabetes mellitus” in Section 6 and Section 7 and in the tables should be read as GBD’s cause label for diabetic-retinopathy-mediated vision loss, not as a claim that diabetes causes vision loss independently of retinal disease; not all people with diabetes mellitus develop diabetic retinopathy, and GBD’s estimate reflects the population-level ocular complication rate, not diabetes prevalence itself. A supplementary severity-by-cause extract described which causes contributed to each severity category, resolving eight of the nine tracked causes to the severity level; only “diabetes and kidney diseases” is not resolved to the severity level, which is immaterial, since that label duplicates “diabetes mellitus” and was excluded from the analysis to avoid double-counting (see Section 6.4).

Reconciling age-group counts. The age-period extracts (below) returned 50 distinct GBD age-band labels in total. Of these, 22 are mutually exclusive and jointly exhaustive fine-grained bands (<1 year, 12–23 months, 2–4, 5–9, 10–14, 15–19, 20–24, 25–29, 30–34, 35–39, 40–44, 45–49, 50–54, 55–59, 60–64, 65–69, 70–74, 75–79, 80–84, 85–89, 90–94, and 95+ years) and were retained for the age-period analysis reported here; the remaining 28 labels are overlapping aggregate bands at coarser granularity (e.g., “0–14 years,” “20+ years,” “70–74 years” duplicated within a wider “70+ years” aggregate) or finer neonatal subdivisions of the under-1 band (0–6 days, 7–27 days, 1–5 months, 6–11 months) that sum to the “<1 year” band already included. These 28 overlapping or redundant labels were excluded from the age-period analysis to avoid double-counting, and the 22 retained bands sum exactly to the “All ages” total in every year checked. The full crosswalk from all 50 downloaded labels to the 22 analytic bands can be reproduced from the replication script and raw files deposited in the OSF repository (see Data Availability Statement).

### 6.4. Procedure

#### 6.4.1. Data Collection

Six extracts were downloaded from the GBD Results Tool, each restricted to Saudi Arabia and to the full 1990–2023 year range, and each fixing four or five of the six filterable GBD dimensions to keep individual extracts within the GBD Results Tool’s per-query row limit: (1) a cause-decomposition extract (Number, Percent, and Rate; all ages; nine causes; aggregate severity; both sexes and by sex); (2) a severity-by-cause extract (Number only; seven causes; five severity categories; all ages; both sexes and by sex); (3) a core trend extract (Number, Percent, and Rate; age-standardised and all-ages; five severity categories; aggregate cause; both sexes and by sex); (4) an age-period extract for YLDs (Number only; approximately 50 GBD age bands; aggregate severity and cause; both sexes combined); (5) an age-period extract for both YLDs and Prevalence (Number only; same age, severity, and cause restrictions); and (6) a diabetes-specific severity extract (Number only; the three diabetes-related causes; four severity categories; all ages; both sexes and by sex), resolving diabetes-attributable burden to the severity level. Age-standardised rates were downloaded directly rather than reconstructed post hoc from Number values, because GBD rates are computed against GBD’s internal population denominators, and independent reconstruction risks methodological mismatch. No age-standardised cause-specific rate extract, and no complete (beyond nine pre-specified causes) cause-hierarchy extract, were obtained; the consequences of this scope restriction for interpretation are stated explicitly wherever cause-specific results are reported, and in Section 8.6. All raw extracts, replication scripts, and analysis output are openly available via the Open Science Framework (OSF; https://osf.io/dn5f6/overview?view_only=be33e1f620c346cc80c19e0d3abc3137, accessed on 31 July 2026).

#### 6.4.2. Cause Selection

The nine causes extracted in the cause-decomposition file were selected a priori as the causes most consistently implicated in the Saudi and regional VI literature reviewed in Section 1 and Section 5 (cataract, glaucoma, uncorrected refractive error, age-related macular degeneration, and diabetes mellitus, together with diabetes mellitus’s type 1 and type 2 sub-components and the overlapping “diabetes and kidney diseases” label). Of these, five are non-overlapping and were carried forward into the composition analysis reported in Section 7.2 (cataract, glaucoma, uncorrected refractive error, age-related macular degeneration, and diabetes mellitus); as reported in Section 7.2, these five causes account for only 44–51% of the total “blindness and vision loss” envelope. This means approximately half of Saudi Arabia’s VI burden is attributable to causes outside this pre-specified set, most plausibly a long tail of less common ocular conditions not individually extracted. Because the complete GBD cause hierarchy for this impairment was not downloaded, the cause-composition findings reported here describe trends among the tracked causes, not the full national cause structure of VI, and every cause-specific claim in Section 7, Section 8 and Section 9 is phrased accordingly.

#### 6.4.3. Data Analysis

All analyses were conducted in R (version 4.5.3) [32] using the dplyr, tidyr, readr, segmented, lmtest, and sandwich packages. The primary trend statistic was the estimated annual percentage change (EAPC), calculated by fitting a log-linear regression of the age-standardised rate on calendar year, EAPC = 100 × (e^β^ − 1), with a trend classified as increasing if the lower bound of the 95% confidence interval exceeded zero, decreasing if the upper bound was below zero, and stable otherwise [25]. EAPC was calculated separately for prevalence and YLDs, for each severity category, and for both sexes combined and by sex.

#### 6.4.4. Uncertainty Propagation and Autocorrelation

Two further checks address whether the ordinary-least-squares (OLS) confidence interval around each EAPC adequately reflects the underlying uncertainty. First, because annual GBD estimates are themselves modelled quantities with their own reported 95% uncertainty interval (the upper/lower bounds supplied with every GBD extract), treating each year’s point estimate as exact, as the OLS regression does by default, understates total uncertainty if that source of variation is not incorporated. GBD uncertainty was therefore propagated via a parametric Monte Carlo procedure: for each series, 2000 replicate series were simulated by resampling each year’s log-transformed value from a normal distribution centred on the reported point estimate with a standard deviation implied by the reported 95% uncertainty interval, the log-linear slope was refit on each replicate, and the 2.5th and 97.5th percentiles of the resulting EAPC distribution were reported as a GBD-uncertainty-propagated confidence interval.

A second, distinct source of understated uncertainty was addressed separately. A Durbin-Watson test was applied to the residuals of each primary EAPC regression to assess serial autocorrelation among consecutive annual estimates, since GBD estimates for adjacent years are unlikely to be statistically independent. This test indicated significant positive autocorrelation in every series tested (Durbin-Watson statistics ranging from 0.12 to 0.70, all *p* < 0.001 except two presbyopia series at *p* < 0.001 as well), meaning OLS standard errors are anti-conservative throughout. Newey-West heteroskedasticity- and autocorrelation-consistent (HAC) standard errors were therefore used to construct a second, autocorrelation-robust confidence interval for every reported EAPC. Because the GBD-uncertainty-propagated interval and the HAC-robust interval address two distinct sources of understated uncertainty and do not always agree, a trend is reported in the main tables and text as confirmed only where the naive OLS, HAC-robust, and GBD-uncertainty-propagated intervals all excluded zero in the same direction, and as suggestive (directionally consistent but not robust to the choice of uncertainty method) otherwise. Point estimates are unaffected by this procedure; only the associated uncertainty and resulting trend classification are affected.

As a sensitivity analysis, segmented (joinpoint-style) regression with Davies’ test for a change in slope [33] (as implemented in the segmented package) was fitted to each series to test whether a single linear trend adequately described the 1990–2023 period or whether a statistically identifiable breakpoint improved model fit. Where a breakpoint was statistically supported, segment-specific EAPCs were additionally calculated separately for the pre- and post-breakpoint periods, since a single 34-year EAPC can obscure a change in pace; these segment-specific results are treated as a substantive part of the results (Section 7.1) rather than only as a sensitivity check, and are interpreted cautiously where a post-breakpoint segment is short (as short as four to five annual points for the most recent breakpoints, discussed below). To characterise the contribution of population ageing to the observed trend, EAPC was additionally calculated on crude (“all ages”) rates and compared against the age-standardised EAPC for the same series; a materially more negative (or less positive) age-standardised trend relative to the crude trend is interpreted as consistent with population ageing masking improvement in age-specific rates, following the logic used in prior GBD ageing-decomposition studies [13], but this comparison is descriptive rather than a formal demographic decomposition, and no specific numerical fraction of the change is attributed to ageing on this basis alone (see Section 7.3 and Section 8.6). Cause-specific contributions were examined descriptively via the percentage-point change in each cause’s share of total burden between 1990 and 2023, together with cause-specific EAPC calculated on crude rates, since an age-standardised cause-specific rate extract was not obtained (see Section 6.4.1); cause-specific EAPCs may therefore reflect both underlying epidemiological change and Saudi Arabia’s changing population structure, and are described as crude throughout. The age distribution of the burden was examined by comparing the percentage change in YLDs and prevalence between 1990 and 2023 across 22 mutually exclusive GBD age bands; because this comparison uses case counts (Number) rather than age-specific rates, an age-specific rate extract requiring GBD population denominators was not obtained in this study, a large percentage change in a given age band can reflect population growth in that band as well as, or instead of, a genuine change in age-specific risk, and Section 7 and Section 8 are worded accordingly.

#### 6.4.5. Sex-Difference Testing

Male and female trends were compared formally, rather than only by inspecting separately estimated EAPCs, by fitting a log-linear regression of rate on year, sex, and their interaction for each series (log(rate) ~ year × sex); the interaction term’s coefficient and an F-test comparing this model against the corresponding main-effects-only model provided a formal test of whether the male and female slopes differ.

#### 6.4.6. Multiple Testing

This study conducted 96 statistical tests in total across five related families (30 primary severity/sex EAPC tests, 10 joinpoint/segmented-regression tests, 10 sex-difference interaction tests, 30 Durbin-Watson autocorrelation checks, and 16 cause-specific EAPC tests). These analyses are exploratory and descriptive rather than confirmatory of a single pre-registered hypothesis, consistent with a repeated cross-sectional trend-characterisation design; no formal multiple-testing correction was applied, and findings are interpreted with reference to effect size and consistency across the three uncertainty-estimation methods described above rather than on the basis of nominal significance alone, in order to guard against over-interpreting isolated significant results arising from the number of comparisons performed.

#### 6.4.7. Ethical Considerations

This study used publicly available, de-identified, aggregate secondary data from the GBD Results Tool (GBD 2023 release) [31]; no individual-level or identifiable data were accessed, and no primary data collection involving human participants was undertaken. Institutional ethical approval was therefore not required. Data use complied with IHME’s free-use data terms, and the GBD 2023 data source is cited in accordance with IHME’s citation requirements [30,31].

## 7. Results

This study set out to answer three questions about the burden of VI in Saudi Arabia between 1990 and 2023: how the burden changed by severity category and sex (Aim 1), which causes among those examined drove that change (Aim 2), and whether population ageing is consistent with an important contribution to that change (Aim 3). Results are presented in that order, one subsection per aim, with the relevant table or figure cited at first mention. Numerical findings are reported descriptively in this section; their interpretation, and any claim about mechanism, is reserved for Section 8.

### 7.1. Aim 1: Overall, Severity-Specific, and Sex-Specific Trends

**Figure 1:**
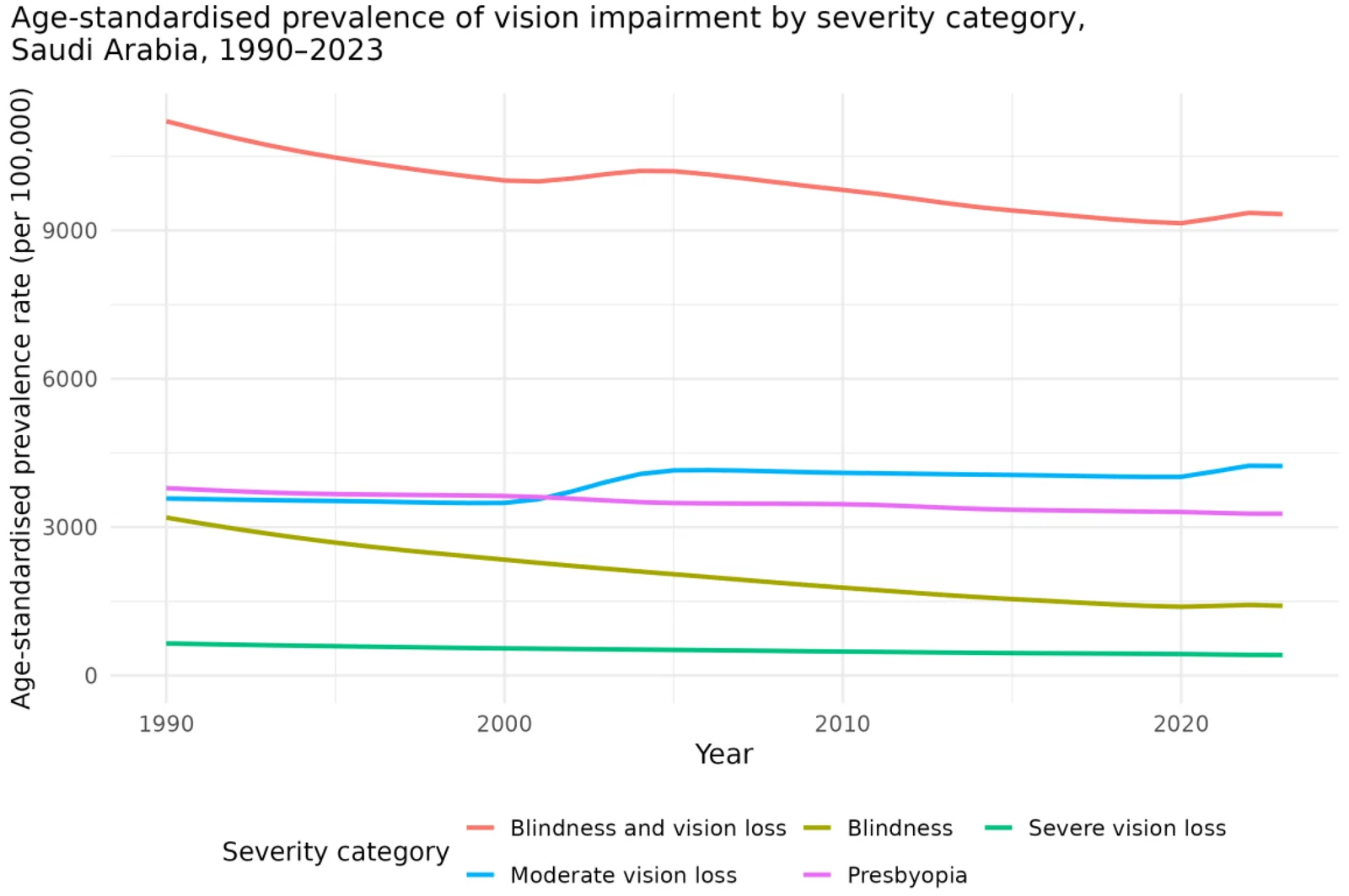
Age-Standardised Prevalence of Vision Impairment by Severity Category, Saudi Arabia, 1990–2023.

Between 1990 and 2023, the age-standardised prevalence of the aggregate “blindness and vision loss” envelope in Saudi Arabia declined in point-estimate terms (EAPC = −0.53%/year), although this specific series was not robust to the choice of uncertainty method (see below), and the age-standardised YLD rate declined more markedly and robustly (EAPC = −1.63%/year). Table 1 breaks this overall figure down by severity category and sex, now reporting three confidence intervals for every estimate: the naive OLS interval used in the original analysis, a Newey-West HAC-robust interval that accounts for the autocorrelation confirmed in every series (Durbin-Watson statistics 0.12–0.70, *p* < 0.001 in 28 of 30 series), and a GBD-uncertainty-propagated interval obtained by Monte Carlo resampling within each year’s reported 95% uncertainty interval. A trend is labelled confirmed only where all three intervals agree on direction, and suggestive where they do not. Nineteen of the 30 severity-by-sex-by-measure series were confirmed; 11 were suggestive only. Age-standardised blindness declined the most steeply of any category and was confirmed decreasing in both sexes separately (prevalence EAPC = −2.60%/year, both sexes; female −2.68%/year; male −2.52%/year), severe VI also declined and was confirmed in all three sex strata (prevalence EAPC = −1.30%/year), and presbyopia declined in point-estimate terms (prevalence EAPC = −0.44%/year) but this trend was suggestive only, not confirmed, once GBD’s own reported uncertainty was propagated into the estimate. Moderate VI moved in the opposite direction and was the only severity category to increase, confirmed for both sexes combined and for males specifically (prevalence EAPC = +0.61%/year, both sexes, confirmed; male +0.70%/year, confirmed), while the female-specific increase (prevalence EAPC = +0.50%/year) and the YLD-scale increase for moderate VI in all sex strata were suggestive only. Figure 1 plots the full 1990–2023 trajectory for each severity category and shows that this divergence was not a recent development: moderate VI overtook the aggregate presbyopia trend in prevalence terms during the early 2000s and has remained the fastest-growing category since.

The sex breakdown in Table 1 shows a pattern across every severity category consistent with a formal test of the difference: the direction of change was identical for males and females, but the magnitude differed in a way that tracked the direction of change itself. Where a category was declining, the decline was somewhat steeper in females (e.g., blindness: −2.68%/year in females vs. −2.52%/year in males; the aggregate envelope: −0.61%/year in females vs. −0.46%/year in males). Where a category was rising, the rise was steeper in males (moderate VI: +0.70%/year in males vs. +0.50%/year in females). A formal sex × year interaction test (Table 2) confirmed a statistically significant difference in slope between males and females for 8 of the 10 measure-by-severity series tested (interaction *p* < 0.05); the two exceptions, where the numerical difference did not reach formal significance, were YLDs for moderate vision loss (interaction *p* = 0.059) and prevalence for severe vision loss (interaction *p* = 0.085), and the corresponding claims below are worded accordingly. No severity category reversed direction between sexes, so the sex difference reported here is one of pace rather than of direction.

The segmented-regression sensitivity analysis, reported in full in Table 3, supported a single statistically identifiable change in slope for most series rather than a strictly linear 34-year trend (Davies’ test *p* < 0.05 for 8 of the 10 measure-by-severity combinations tested). Breakpoints clustered around 1995–1998 for the aggregate envelope and for severe VI, and around 2008 for moderate VI; blindness showed a later breakpoint, in 2019, for both prevalence and YLDs. Presbyopia was the exception, showing no statistically identifiable breakpoint (*p* = 0.27–0.33), matching the smoother, closer-to-linear decline visible for that category in Figure 1. These breakpoints mark where the trend’s slope changed, not a discrete event that caused it, and should be read descriptively. Because these breakpoints materially change how the 1990–2023 trend should be interpreted, Table 4 reports the segment-specific EAPC before and after each supported breakpoint, rather than treating the breakpoint as a secondary check on the single 34-year EAPC already reported in Table 1.

A pattern common to blindness and moderate VI in Table 4 warrants direct statement, since it qualifies the headline single-period EAPCs in Table 1. For blindness, the strong 1990–2019 decline (prevalence EAPC = −2.77%/year) was not sustained in the 2019–2023 segment, whose point estimate is positive (+0.29%/year) but whose confidence interval spans zero; the same pattern holds for blindness YLDs. For moderate VI, the strong 1990–2008 increase (prevalence EAPC = +1.01%/year) likewise gives way to a 2008–2023 segment whose point estimate is small and not statistically distinguishable from zero (+0.09%/year). Read together with the robustness verdicts in Table 1, this indicates that both the decades-long decline in blindness and the decades-long rise in moderate VI were concentrated in the earlier part of the study period, and that the post-breakpoint segments, four to fifteen years in length, do not yet provide statistical evidence that either trend is continuing at its earlier pace; they are equally consistent with a plateau. Given how short the most recent segments are (as few as five annual points), this should be read as an indication that recent-period trends warrant continued monitoring in future GBD rounds, not as evidence that either trend has reversed.

### 7.2. Aim 2: Cause-Specific Contributions (Among Tracked Causes)

Nine pre-specified causes were examined. Five were non-overlapping (cataract, glaucoma, uncorrected refractive error, age-related macular degeneration [AMD], and diabetes mellitus, the last combining its type 1 and type 2 sub-components), while “diabetes and kidney diseases” was numerically identical to “diabetes mellitus” in this extract and was excluded from summed shares to avoid double-counting. Together, the five non-overlapping causes accounted for 44–51% of the aggregate envelope’s Number (prevalence: 43.6% in 1990, 44.8% in 2023; YLDs: 50.7% in 1990, 50.3% in 2023), so roughly half of the total burden is attributable to causes outside this pre-specified set. Table 5 therefore describes the relative composition among the five tracked causes, not the full burden envelope, and every finding in this subsection should be read with the phrase “among tracked causes” implied even where not repeated in full; this scope restriction is carried forward as a stated limitation in Section 8.6.

As Table 5 shows, uncorrected refractive error had the largest prevalence share of the five tracked causes throughout the study period, and that share grew further, though the underlying crude-rate increase was suggestive only once autocorrelation-robust uncertainty was taken into account, and should not be described as a statistically clear trend. Diabetes mellitus showed the largest relative gain of any tracked cause: its share of tracked prevalence roughly quadrupled, on the back of the steepest crude-rate increase in the entire cause set, and this finding was confirmed under every uncertainty specification tested. Cataract and glaucoma moved the other way, both falling in relative share and in crude rate over the same period, though only the glaucoma decline was confirmed robust; the cataract prevalence decline was directionally consistent but suggestive only, and AMD’s share and rate both declined modestly as well, also suggestive only. The YLD measure told a broadly similar story to prevalence for four of the five causes, with one exception worth flagging directly: uncorrected refractive error gained YLD share (36.2% to 50.0%) even though its own crude YLD rate was statistically stable (EAPC = +0.07%/year, 95% CI [−0.03, 0.16]). That gain in relative share is therefore an artefact of the other causes’ YLD shares falling around it—cataract’s YLD share fell by 14.5 percentage points and glaucoma’s by 8.5—rather than evidence that uncorrected refractive error itself became more disabling.

The severity-by-cause breakdown showed that cataract’s contribution to blindness fell from 18.0% of blindness-specific prevalence in 1990 to 12.3% in 2023, while uncorrected refractive error dominated moderate VI throughout the period (35.9% in 1990, 33.8% in 2023) and was the attributed cause of presbyopia by construction of the GBD cause hierarchy; presbyopia’s inclusion as a severity category alongside pathological causes such as cataract, glaucoma, and diabetic retinopathy follows GBD’s own impairment classification (see Section 6.3) rather than an editorial choice by the present authors, and its very high population prevalence (nearly universal among adults over approximately 45 years) together with its comparatively small and, per Table 1, statistically fragile trend is precisely why it is reported separately from, and not aggregated with, the three acuity-based severity categories throughout this manuscript.

Table 6 shows the severity distribution of diabetes-attributable VI, addressing which severity category carries this burden within Aim 2; this distribution is reported using case counts (Number), since a rate-based, severity-specific breakdown for diabetes was not obtainable from the available extracts, and is therefore descriptive of composition rather than a trend statistic in its own right (see Section 8.6). The answer differs depending on whether prevalence or YLDs are examined, and that difference is itself informative. In prevalence terms, diabetes-attributable VI is dominated by moderate VI, and increasingly so (56.3% of diabetes-attributable prevalence in 1990, rising to 63.5% in 2023), while its contribution to severe VI fell by roughly half over the same period (16.9% to 8.9%). In YLD terms, however, the picture inverts: blindness accounts for the largest and a growing share of diabetes-attributable YLDs (50.6% in 1990, rising to 58.9% in 2023). This divergence follows directly from how GBD constructs the YLD measure (see Section 6.3): diabetes causes many more cases of moderate VI than of blindness, but each case of diabetes-attributable blindness carries a far higher disability weight, so blindness dominates the disability-adjusted (YLD) measure even while it is a minority of the case count (prevalence) measure. This is a definitional consequence of disability weighting, not evidence that individual patients progress from one severity category to another; the data used here do not follow individuals over time and cannot establish within-person progression (see Section 8).

### 7.3. Aim 3: Age Distribution and the Ageing Comparison

**Figure 2:**
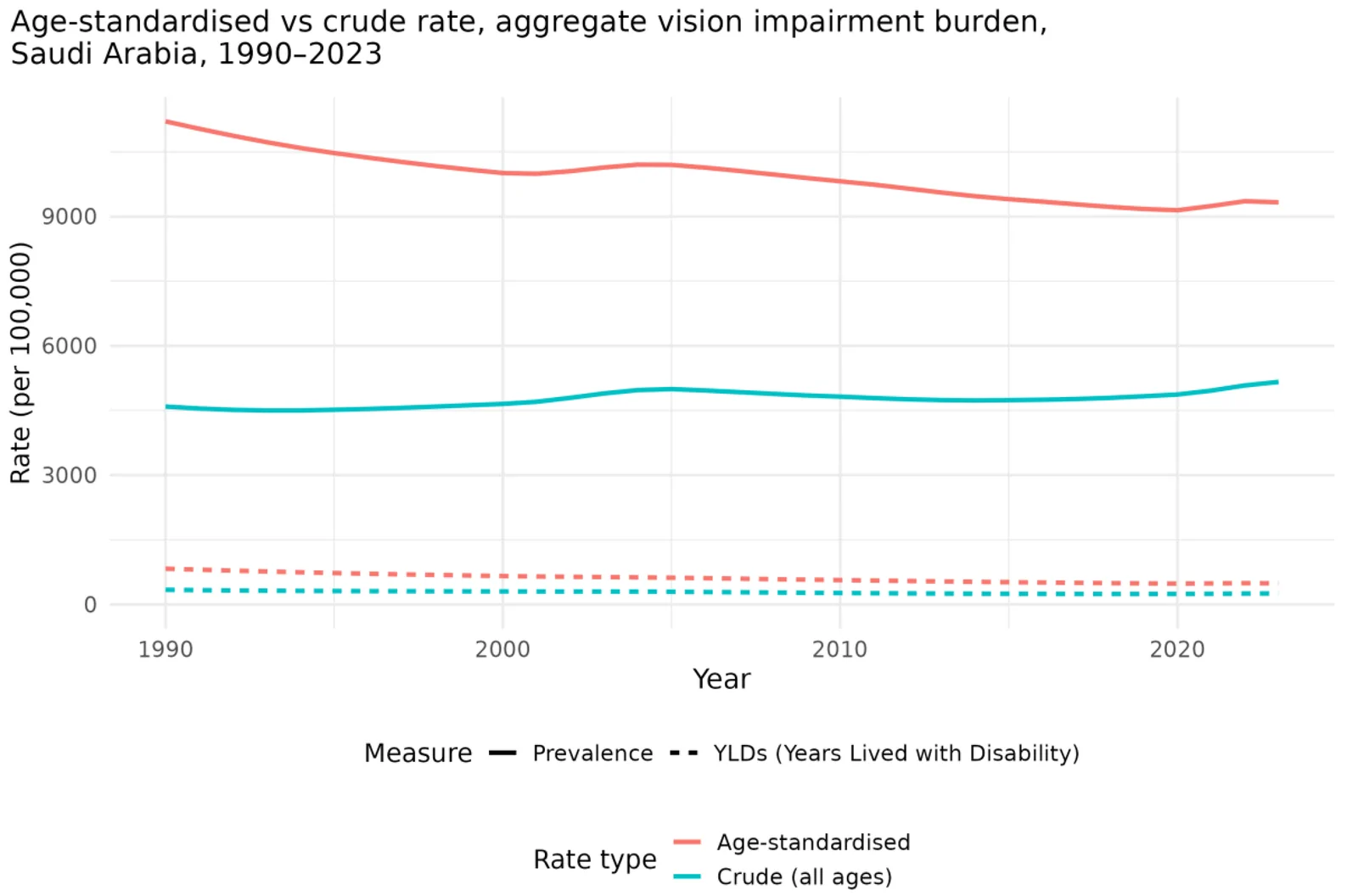
Age-Standardised Versus Crude Rate, Aggregate Vision Impairment Burden, Saudi Arabia, 1990–2023, Prevalence and YLDs.

Figure 2 plots the age-standardised and crude trend side by side for the aggregate envelope, and the two lines diverge in a way consistent with a real ageing effect, though this comparison is descriptive and does not itself decompose the change into a specific ageing-attributable percentage (see Section 6.4 and Section 8.6). For aggregate prevalence, the age-standardised trend was decreasing in point-estimate terms (EAPC = −0.53%/year, suggestive only per Table 1) while the crude, all-ages trend was increasing (EAPC = +0.28%/year, 95% CI [0.19, 0.37]): a gap of roughly 0.8 percentage points per year consistent with Saudi Arabia’s changing age structure contributing to this difference, rather than to any change in age-specific risk. YLDs showed the same pattern (age-standardised EAPC = −1.63%/year vs. crude EAPC = −1.02%/year). For moderate VI, both the age-standardised and the crude rate increased, but the crude increase was around twice as large as the age-standardised one (prevalence: +1.32%/year crude vs. +0.61%/year age-standardised), a gap consistent with population ageing contributing part of the observed rise in moderate VI, though this study’s descriptive comparison cannot, on its own, partition the remainder into a specific attributable fraction for genuine age-specific risk change. Blindness stood apart from this pattern: its age-standardised and crude trends were similar in both direction and size, both strongly decreasing, consistent with a reduction in age-specific blindness risk that owes comparatively little to demographic change, though again on a descriptive rather than formally decomposed basis.

**Figure 3:**
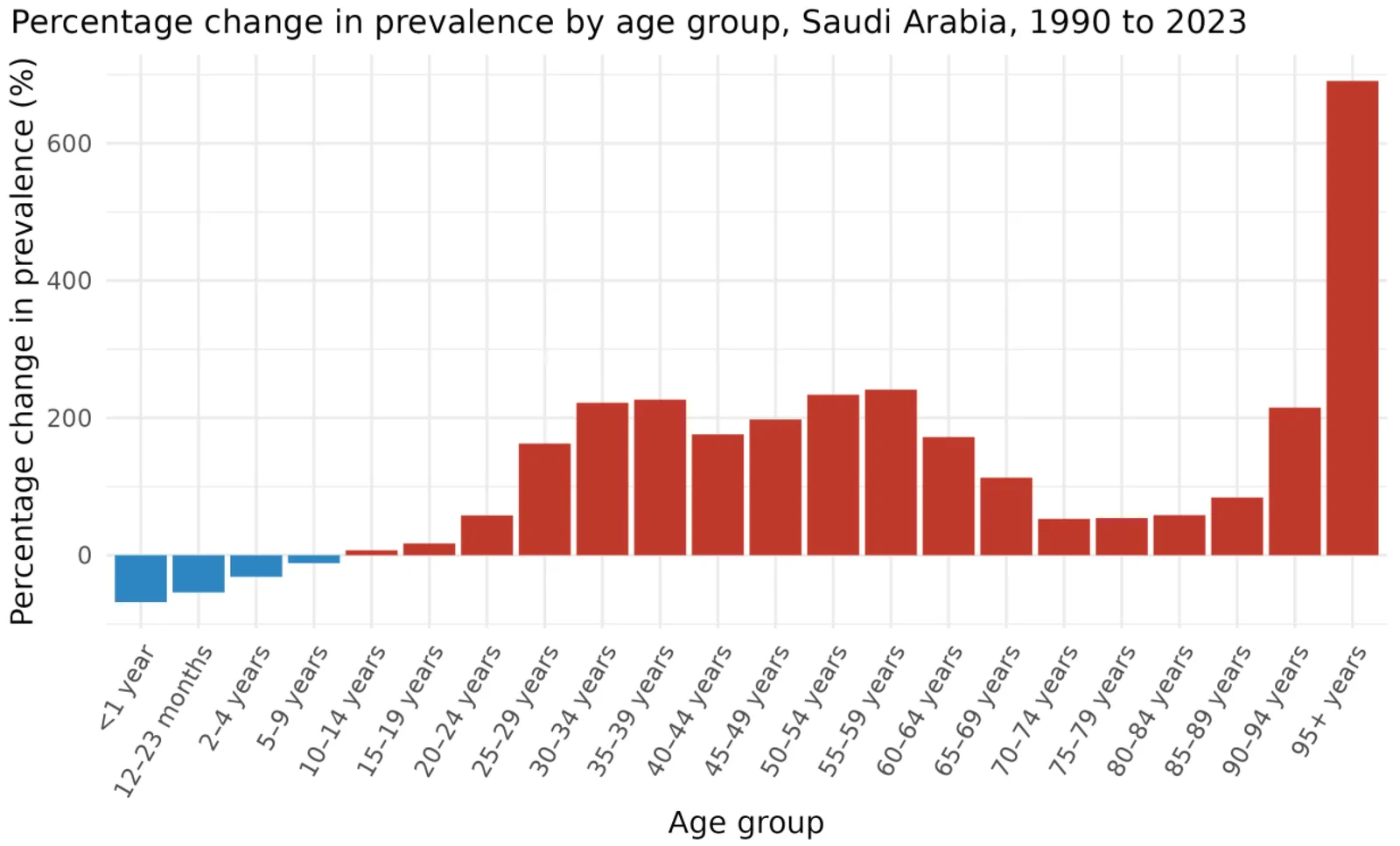
Percentage Change in Prevalence by Age Group, Saudi Arabia, 1990 to 2023.

**Figure 4:**
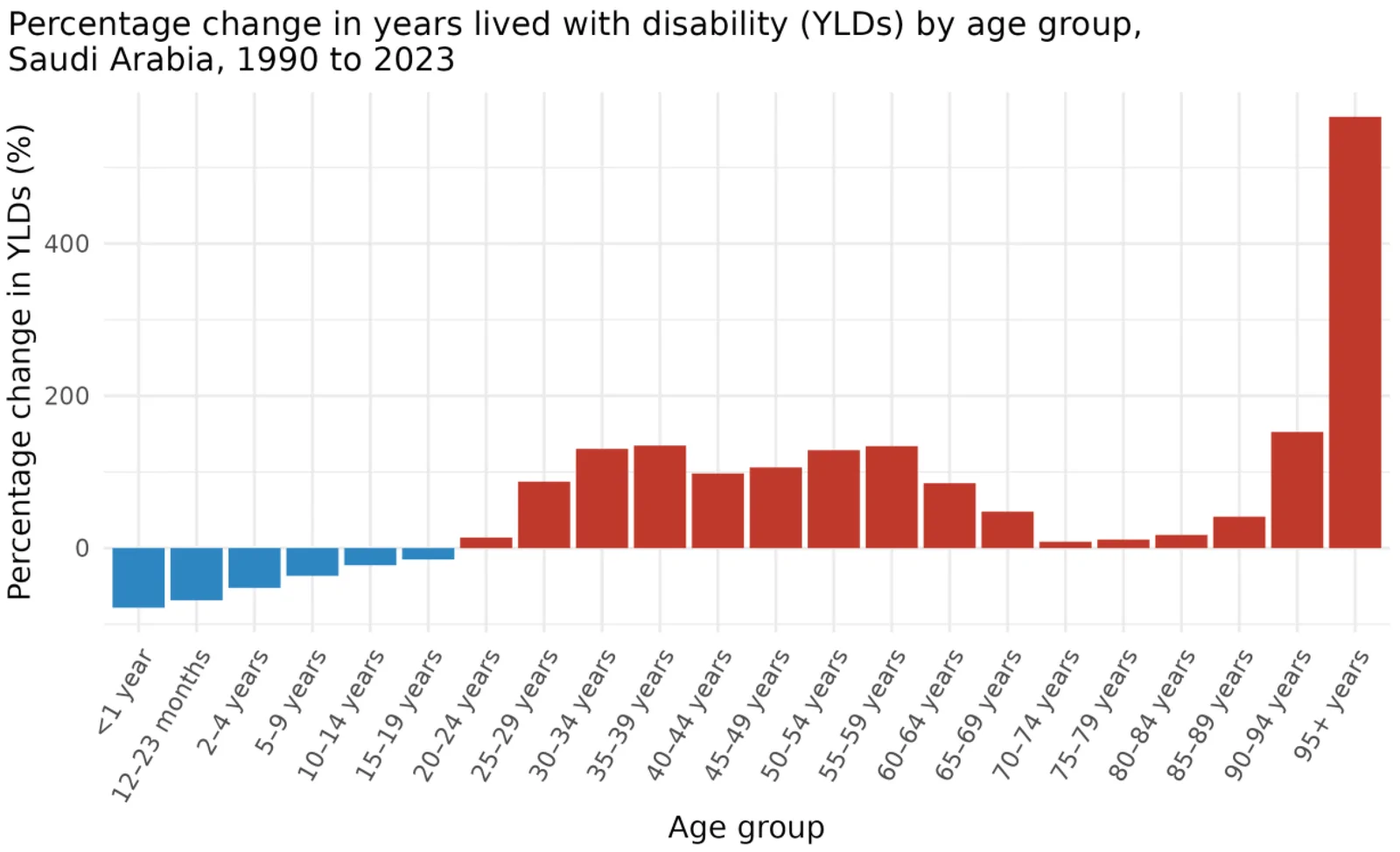
Percentage Change in Years Lived with Disability by Age Group, Saudi Arabia, 1990 to 2023.

Figure 3 shows the percentage change in prevalence between 1990 and 2023 across 22 mutually exclusive age bands, and Figure 4 shows the equivalent percentage change in YLDs; both figures plot case counts (Number), not age-specific rates, since an age-specific rate extract was not obtained (see Section 6.4), so the patterns below describe how the number of people affected in each age band has changed, not necessarily how an individual’s risk within that age band has changed. With that caveat, every age band under 20 years fell, in some cases sharply (e.g., −68.3% in infants under one year, −31.4% in the 2–4 years band), while among adults under 90 years the largest proportional increases in case counts were concentrated not in the oldest groups but in the 30–59 years range (prevalence: +222% to +241%; YLDs: +98.1% to +134.7%). The 95 years and older band showed the single largest percentage increase of any age group in both measures (prevalence: +690.8%; YLDs: +566.6%), but this reflects a very small 1990 baseline (181 prevalent cases; 16 YLDs) rather than a substantively large shift in absolute burden, and should be interpreted with caution alongside the more robust working-age pattern. Outside this small, high-variance oldest band, the age-period pattern is consistent across both measures: the increases are concentrated in working-age adults rather than in the oldest age groups, which is directionally consistent with the diabetes-related mechanism examined in Section 8 rather than with an ageing-driven mechanism operating uniformly across all older age groups; because these are case counts rather than rates, this pattern is also consistent with faster population growth among working-age adults over the study period, and Section 8 weighs both explanations rather than treating the case-count pattern alone as decisive.

## 8. Discussion

This study found that between 1990 and 2023, Saudi Arabia’s age-standardised burden of blindness and severe VI declined markedly and robustly, while moderate VI rose robustly overall and among males specifically, diabetes mellitus (the GBD label under which diabetic-retinopathy-mediated vision loss is coded; see Section 6.3) increased faster than any other tracked cause and did so under every uncertainty specification tested, and the largest proportional increases in case counts fell among working-age adults rather than concentrating in older adults alone. Some of the more specific claims made in earlier analyses of this dataset, however, do not survive the additional robustness checks reported here, and this section is organised to keep that distinction explicit throughout: what the data show, what is a plausible explanation consistent with but not established by the data, and what remains a hypothesis for future research.

### 8.1. Consistency with Regional and Global GBD Evidence

The core pattern observed here —falling blindness alongside rising moderate VI—mirrors the most direct available comparator, a MENA-wide GBD 2019 analysis reporting the same divergent trend across the region [24], and it echoes the global pattern in which age-standardised blindness has declined even as overall vision-loss burden rises [25]. The present analysis extends this evidence in two respects. First, it isolates Saudi Arabia specifically, whereas Qaddoumi et al. [24] and the earlier MENA analysis by Bahremani et al. [20] pool 20 or more countries of markedly different socio-demographic profiles and diabetes prevalence; the Saudi-specific EAPCs reported here (e.g., blindness prevalence EAPC = −2.60%/year, confirmed robust) provide a national estimate that a pooled regional figure cannot. Second, it extends the observation window to 2023, four years beyond the most recent prior regional GBD analysis [20]. This extension yields a specific new finding of its own, evident directly in the segmented-regression results: both the blindness decline and the moderate-VI rise are concentrated in the earlier part of the 1990–2023 window (pre-2019 and pre-2008 respectively), and the post-breakpoint segments captured only by extending to 2023 are not yet statistically distinguishable from a plateau. A 2019 cut-off could not have revealed this, and it is arguably the single most policy-relevant observation to emerge from the fuller series: continued decline in blindness should not be assumed to be automatic, and continued monitoring in future GBD rounds is warranted rather than continued extrapolation from the 1990–2019 pace.

### 8.2. The Diabetes-Driven Shift

The steep rise in diabetes-attributable VI rates (crude EAPC ≈ +4.47%/year, confirmed under every uncertainty specification tested, the fastest of any tracked cause) fits Saudi Arabia’s well-documented type 2 diabetes epidemic. Population-based surveys report that Saudi adult diabetes prevalence rose from 10.6% in 1989 to 32.1% in 2009 [26], with more recent systematic reviews placing current prevalence at 21–32% and projecting further increases [3,4], and a 2024 systematic review estimating that diabetic retinopathy affects approximately 31% of Saudi adults with diabetes (24% among those with type 2 diabetes specifically), with glycaemic control and diabetes duration as the strongest predictors [2]. This echoes global GBD-based evidence that diabetic retinopathy is an increasingly important driver of VI specifically among the working-age population [17] and that the global burden of diabetes-attributable blindness and vision loss is projected to continue rising to 2050 [16]. Consistent with Epidemiologic Transition Theory [6], this pattern is compatible with a national disease profile continuing to shift from the historically dominant, largely avoidable causes of blindness (cataract, trachoma, uncorrected refractive error) toward causes tied to chronic disease and population ageing; the present, ecological, cause-decomposition design cannot itself test the transition mechanism, but the observed divergence, declining cataract- and glaucoma-attributable rates alongside a robustly rising diabetes-attributable rate, is the pattern the theory would predict at this stage of Saudi Arabia’s demographic and epidemiological development.

A second, age-specific pattern in the data points toward the same mechanism. The age-period findings reported here—with the largest proportional increases in case counts concentrated in the 30–59 years age range rather than in the oldest age groups—align with this diabetes-driven, working-age-skewed mechanism rather than with an ageing-driven mechanism alone, though, because these age-period findings are based on case counts rather than age-specific rates, faster working-age population growth over the study period offers a second, not mutually exclusive explanation that this study’s data cannot rule out, and offer a Saudi-specific illustration of a pattern so far mainly documented globally or in a China-specific GBD analysis reporting a rising age-standardised prevalence and EAPC for type 2 diabetes-attributable blindness [34].

Table 6 sharpens this picture further. Most diabetes-attributable prevalence in Saudi Arabia sits in the moderate VI category, and increasingly so, while the YLD burden is dominated by blindness. As explained in Section 7.2, this divergence follows directly from GBD’s disability-weighting construction of the YLD measure rather than from within-person clinical progression, since this cross-sectional annual series does not follow individuals over time and cannot establish that a given patient moves from moderate VI to severe VI to blindness. With that caveat stated explicitly, the compositional pattern, a large and growing pool of people with diabetes-related moderate VI alongside a smaller but highly disabling pool with diabetes-related blindness, is compatible with a clinical hypothesis worth stating for future research rather than as a finding this study establishes: that diabetic retinopathy screening and laser or anti-vascular endothelial growth factor (anti-VEGF) treatment may be intercepting some cases before progression to blindness, while a much larger volume of earlier-stage diabetic eye disease continues to accumulate. Screening and treatment variables were not measured in this GBD-based analysis, and this explanation should be read as a hypothesis motivated by the compositional pattern, not as a mechanism this study has tested. It also gives the “diabetes prevention and retinopathy screening” recommendation made below a sharper target: if this hypothesis is correct, the priority would not primarily be to prevent blindness in patients who already have advanced disease, but to intervene earlier, while most affected patients are still in the moderate VI category—though this remains a hypothesis for future clinical research to test directly, not a conclusion this ecological analysis can establish on its own.

### 8.3. Divergence from the AMD Literature

In contrast to global and regional GBD analyses reporting rising AMD-attributable burden [14,15], the present analysis found a modest decline in AMD-attributable prevalence and rate in Saudi Arabia over the study period, though this specific finding was suggestive only, not confirmed, once autocorrelation-robust and GBD-uncertainty-propagated intervals were applied (Table 5). This divergence may reflect Saudi Arabia’s comparatively young population structure relative to the higher-income, older-population settings that dominate the global AMD-burden literature, though the present ecological, cause-decomposition design cannot adjudicate between a genuine cohort effect, a data or modelling artefact, and simple estimation uncertainty given the modest confidence in this particular trend; this represents a specific avenue for future Saudi-focused AMD research rather than a finding this study can explain further.

### 8.4. Sex Differences

Table 1 and the formal interaction test in Table 2 show that males and females moved in the same direction on every severity category but at different speeds, with the difference formally confirmed for 8 of the 10 series tested: declines were steeper in females, and the rise in moderate VI was steeper in males (both confirmed). The two series where the sex difference did not reach formal significance, severe vision loss prevalence and moderate vision loss YLDs, are described here only as numerically consistent with the broader pattern, not as confirmed sex differences in their own right. This pattern lines up with the diabetes evidence summarised above, in which Saudi male diabetes prevalence has risen faster than female prevalence (0.8% vs. 0.6% per year) [26]. A faster-rising, male-skewed risk factor matches the faster-rising, male-skewed increase in moderate VI observed here, though the ecological design of this study means the two findings can only be described as compatible, not causally linked, and the Andersen Behavioral Model [27] offers one, but only one, plausible framework for why: if Saudi men have later diagnosis, lower screening participation, or a longer average duration of untreated diabetes than Saudi women, whether from differences in enabling resources (service access, work schedules), predisposing characteristics, or perceived need, that would be compatible with a male-skewed rise in diabetes-attributable VI. None of the model’s predisposing, enabling, or need constructs were measured here, so this is offered as a hypothesis the present findings motivate, not as a mechanism this study demonstrates.

The 2023 diabetes-severity breakdown adds a further, more specific sex pattern. Among males, a larger share of diabetes-attributable prevalence fell into blindness than among females (30.4% vs. 23.8%), while females carried a larger share in moderate VI (66.5% vs. 61.3%). The same male-skew toward the more severe end held for YLDs, and more strongly (blindness share of diabetes YLDs: 62.5% in males vs. 53.4% in females). Considered alongside the confirmed male-skewed EAPC for moderate VI, this is compatible with Saudi males accumulating a disproportionate share of diabetes-attributable VI at the more severe end, which in turn is compatible with later diagnosis, later screening uptake, or a longer average duration of untreated diabetes among Saudi men. This ecological analysis can raise that question, motivated by the Andersen model’s enabling- and need-related constructs, but cannot resolve it; a study measuring screening uptake, diagnosis timing, or treatment access directly, by sex, would be needed to test it.

### 8.5. The Role of Population Ageing

The divergence between age-standardised and crude EAPCs for the aggregate envelope (age-standardised: −0.53%/year, suggestive only; crude: +0.28%/year) indicates that Saudi Arabia’s changing age structure is masking a genuine improvement in age-specific VI rates that the crude figures alone would not reveal, a pattern that fits the general GBD finding that population ageing is a major structural driver of rising absolute vision-loss burden even where age-specific risk is falling [13]. However, this study’s descriptive crude-versus-age-standardised comparison is not a formal demographic decomposition, and, per Section 3 and Section 8.6, this manuscript does not assign a specific numerical fraction of the change in moderate VI to ageing; the gap between the crude EAPC (+1.32%/year) and the age-standardised EAPC (+0.61%/year, confirmed increasing) for moderate VI is consistent with population ageing accounting for part of the observed rise, with the remainder plausibly reflecting genuine increases in age-specific risk, but this study’s methods cannot partition that remainder precisely—indicates that ageing alone is unlikely to fully account for Saudi Arabia’s changing burden; the diabetes epidemic operating across working-age adults is at least as plausible a contributor in this national context as the ageing effect that dominates the global GBD ageing literature.

### 8.6. Limitations

Several limitations qualify these findings; each is stated explicitly below, along with what it does, and does not, constrain. First, the nine pre-specified causes examined covered only 44–51% of the total “blindness and vision loss” envelope, so the reported cause shares describe relative composition among tracked causes rather than the full burden; this means the study can identify which tracked cause is changing fastest, but cannot establish that these tracked causes explain the overall national change, since roughly half of the burden lies outside them; a future extraction of GBD’s complete cause list for this impairment would allow a more exhaustive decomposition. Second, the diabetes-by-severity breakdown (Table 6) is available only for Number, not Rate, so it is descriptive (proportional shares) rather than a trend statistic in its own right, meaning it describes how diabetes-attributable burden is distributed across severity but not whether that distribution’s trend reflects rate change or population growth; a rate-based severity-specific EAPC for diabetes was not calculable from the available data. Third, as with all GBD-based analyses, the underlying estimates are themselves modelled from heterogeneous, geographically uneven primary data sources [29], and comparisons against the fragmented Saudi survey literature summarised in Section 1 should be treated as complementary rather than a validation of GBD’s Saudi-specific modelling assumptions. A further set of limitations concerns the study’s design rather than its measurement choices. Fourth, this is an ecological, repeated cross-sectional design; it cannot attribute individual-level causation between diabetes, ageing, and VI risk, and the joinpoint breakpoints reported in Section 7.1 should be read as descriptive markers of where trend slope changed rather than as evidence of discrete causal events.

Fifth, the age-period analysis (Aim 3, Figure 3 and Figure 4) is based on case counts rather than age-specific rates, because an age-specific rate extract requiring GBD population denominators was not obtained; this means the concentration of increases among working-age adults could reflect population growth in that age band, genuine risk change, or both, and this study’s data cannot separate the two. Sixth, the crude-versus-age-standardised comparison used to characterise the ageing effect (Aim 3) is descriptive, not a formal demographic decomposition into population growth, changing age structure, and age-specific rate change; no specific numerical ageing-attributable fraction is reported for this reason, and any future analysis wishing to report one would need a formal decomposition method rather than this comparison. 

A final set of limitations concerns the statistical and construct-coverage boundaries of this analysis. Seventh, although Newey-West HAC-robust and GBD-uncertainty-propagated confidence intervals address autocorrelation and input-estimate uncertainty respectively, no method used here jointly models both sources of uncertainty at once, so the “confirmed” trends in Table 1 and Table 5 should still be read as a conservative, not exhaustive, robustness check. Eighth, 96 statistical tests were conducted across five related families without formal multiple-testing correction (see Section 6.4.6); findings are interpreted with reference to consistency across the three uncertainty-estimation methods rather than on nominal significance alone, but isolated results, particularly among the “suggestive only” trends, should be treated with corresponding caution. Ninth, no screening, diagnosis-timing, treatment, or health-service-utilisation variable was measured in this analysis; the Andersen Behavioral Model-motivated explanations offered above for sex differences, and the screening/treatment explanation offered for the diabetes severity-distribution pattern, are hypotheses for future research, not findings this study demonstrates.

### 8.7. Implications

These findings suggest that Saudi Arabia’s vision-health policy priorities should extend beyond the historically dominant focus on cataract surgical coverage and trachoma elimination—both showing confirmed favourable trends—toward diabetic retinopathy screening and diabetes prevention among working-age adults, and toward improving access to refractive correction and low-vision services for the growing population living with moderate VI, which by definition falls short of the blindness threshold that has historically attracted the most clinical and policy attention. Given that Saudi-specific screening uptake has independently been reported at only 9.1% in a 2025 primary-care survey [5], and given the segmented-regression finding that the post-2019 blindness trend and post-2008 moderate-VI trend are not yet statistically distinguishable from a plateau, monitoring rather than assuming continued improvement is itself a policy-relevant implication of this study.

## 9. Conclusions

Between 1990 and 2023, Saudi Arabia’s burden of vision impairment underwent a marked compositional shift rather than a uniform decline, and several of this shift’s components are robust to alternative, more conservative ways of estimating uncertainty, while some are not. Blindness and severe vision loss fell substantially and robustly in age-standardised terms, though the decline was concentrated before an identifiable breakpoint around 2019, after which the trend is not yet statistically distinguishable from a plateau. Moderate vision loss rose robustly overall and among males specifically, again concentrated before a breakpoint around 2008. Diabetes mellitus (GBD’s label for diabetic-retinopathy-mediated vision loss) increased faster than any other tracked cause and did so robustly under every uncertainty specification applied, concentrated disproportionately among working-age adults in case-count terms. Population ageing’s contribution to these patterns is descriptively consistent with an important role but was not formally decomposed into a numerical fraction in this study. These findings extend prior MENA-regional GBD evidence to the national, Saudi-specific level and to the most recent year available in the GBD 2023 round, and are compatible with Epidemiologic Transition Theory’s account of a shifting disease profile at Saudi Arabia’s stage of demographic and epidemiological development.

On the basis of these findings, and consistent with what the data support, the following are offered as priorities for Saudi Arabia’s vision-health policy and for future research: (1) diabetic retinopathy screening and diabetes prevention should be prioritised for working-age adults specifically, not only older adults; (2) low-vision rehabilitation and refractive-correction services should be expanded to meet the growing population living with moderate, rather than only severe, vision impairment; (3) national VI surveillance should differentiate severity categories rather than reporting blindness alone, since blindness and moderate VI are moving in different directions; (4) the post-2019 blindness trend and post-2008 moderate-VI trend should be monitored in future GBD rounds rather than assumed to continue at their earlier pace; (5) a future extraction of GBD’s complete cause hierarchy for this impairment, and of age-standardised cause-specific rates, would allow the roughly half of the burden currently outside the tracked-cause set to be characterised; (6) a formal demographic decomposition (separating population growth, changing age structure, and age-specific rate change) would allow a specific ageing-attributable fraction to be reported, which this study’s descriptive comparison could not provide; (7) direct measurement of diabetic-retinopathy screening uptake, diagnosis timing, and treatment access, stratified by sex, would allow the sex-difference and screening hypotheses raised in Section 8 to be tested rather than only motivated; and (8) an age-specific rate extract, requiring GBD population denominators, would allow the working-age concentration reported here to be distinguished from population growth in that age band. Future work pursuing these recommendations would further refine Saudi Arabia’s vision-health policy priorities beyond what this study, on its own, can establish.

## Figures and Tables

**Table 1:** Age-Standardised Estimated Annual Percentage Change (EAPC) in Prevalence and YLD Rate, by Severity Category and Sex, Saudi Arabia, 1990–2023, With Three Confidence-Interval Methods.

Severity Category	Sex	Measure	EAPC	HAC-Robust 95% CI	GBD-Uncertainty-Propagated 95% CI	Robustness Verdict
Blindness and vision loss (aggregate)	Both	Prevalence	−0.53	−8.27, 7.86	−0.74, −0.32	Suggestive only
Blindness and vision loss (aggregate)	Both	YLD	−1.63	−1.87, −1.39	−2.18, −1.05	Confirmed decreasing
Blindness and vision loss (aggregate)	Female	Prevalence	−0.61	−1.21, −0.01	−0.84, −0.38	Confirmed decreasing
Blindness and vision loss (aggregate)	Male	Prevalence	−0.46	−10.78, 11.06	−0.68, −0.23	Suggestive only
Blindness	Both	Prevalence	−2.60	−2.93, −2.27	−2.89, −2.31	Confirmed decreasing
Blindness	Female	Prevalence	−2.68	−2.97, −2.39	−2.96, −2.39	Confirmed decreasing
Blindness	Male	Prevalence	−2.52	−2.91, −2.12	−2.81, −2.24	Confirmed decreasing
Blindness	Both	YLD	−2.56	−2.88, −2.24	−3.25, −1.89	Confirmed decreasing
Severe vision loss	Both	Prevalence	−1.30	−1.42, −1.18	−1.48, −1.14	Confirmed decreasing
Severe vision loss	Both	YLD	−1.26	−1.35, −1.17	−1.86, −0.65	Confirmed decreasing
Moderate vision loss	Both	Prevalence	+0.61	0.22, 1.00	0.47, 0.75	Confirmed increasing
Moderate vision loss	Female	Prevalence	+0.50	−0.86, 1.88	0.36, 0.64	Suggestive only
Moderate vision loss	Male	Prevalence	+0.70	0.33, 1.07	0.55, 0.85	Confirmed increasing
Moderate vision loss	Both	YLD	+0.64	0.23, 1.04	−0.16, 1.47	Suggestive only
Presbyopia	Both	Prevalence	−0.44	−0.50, −0.38	−1.02, 0.12	Suggestive only
Presbyopia	Both	YLD	−0.42	−0.45, −0.39	−1.73, 0.93	Suggestive only

Note. EAPC = estimated annual percentage change, log-linear regression of the age-standardised rate on calendar year (point estimate unaffected by CI method). HAC = Newey-West heteroskedasticity- and autocorrelation-consistent standard errors. OLS = ordinary least squares; CI = confidence interval; YLD = years lived with disability; GBD = Global Burden of Disease. “Confirmed” requires the naive OLS interval (the full 30-row table, including all sex strata and the naive interval, is available in the OSF repository; see Data Availability Statement), the HAC-robust interval, and the GBD-uncertainty-propagated interval to agree on direction; “suggestive only” means at least one alternative method’s interval included zero. Source: output/eapc_severity_agestd_COMBINED.csv.

**Table 2:** Formal Test of Male-Versus-Female Trend Differences (Sex × Year Interaction), Age-Standardised Rate, 1990–2023.

Severity Category	Measure	Female EAPC	Male EAPC	Interaction *p*	Difference Confirmed?
Blindness and vision loss (aggregate)	Prevalence	−0.61	−0.46	< 0.001	Yes
Blindness and vision loss (aggregate)	YLD	−1.70	−1.56	0.016	Yes
Blindness	Prevalence	−2.68	−2.52	0.022	Yes
Blindness	YLD	−2.64	−2.49	0.034	Yes
Severe vision loss	Prevalence	−1.27	−1.31	0.085	No
Severe vision loss	YLD	−1.22	−1.28	0.010	Yes
Moderate vision loss	Prevalence	+0.50	+0.70	0.047	Yes
Moderate vision loss	YLD	+0.53	+0.72	0.059	No
Presbyopia	Prevalence	−0.48	−0.40	< 0.001	Yes
Presbyopia	YLD	−0.45	−0.39	< 0.001	Yes

Note. Interaction *p*-value from an F-test comparing a log(rate) ~ year × sex model against the corresponding main-effects-only model. EAPC = estimated annual percentage change; YLD = years lived with disability. Source: output/sex_difference_test.csv.

**Table 3:** Segmented-Regression Sensitivity Check: Davies’ Test for a Change in Slope, Age-Standardised Rate, Both Sexes, 1990–2023.

Series	Measure	Estimated Breakpoint Year	Davies’ Test *p*	Breakpoint Statistically Supported?
Blindness and vision loss (aggregate)	Prevalence	1995	0.005	Yes
Blindness and vision loss (aggregate)	YLDs	2019	< 0.001	Yes
Blindness	Prevalence	2019	< 0.001	Yes
Blindness	YLDs	2019	< 0.001	Yes
Severe vision loss	Prevalence	1998	< 0.001	Yes
Severe vision loss	YLDs	1998	< 0.001	Yes
Moderate vision loss	Prevalence	2008	0.008	Yes
Moderate vision loss	YLDs	2008	0.007	Yes
Presbyopia	Prevalence	2017	0.334	No
Presbyopia	YLDs	1992	0.269	No

Note. A statistically supported breakpoint (Davies’ test *p* < 0.05) indicates that a two-segment model fits the 1990–2023 series better than a single linear trend; it does not identify a cause. YLD = years lived with disability. Source: output/joinpoint_sensitivity.csv.

**Table 4:** Segment-Specific EAPC Before and After Each Statistically Supported Breakpoint, Age-Standardised Rate, Both Sexes.

Series	Measure	Segment	EAPC	95% CI
Blindness and vision loss (aggregate)	Prevalence	1990–1995	−1.35	−2.00, −0.71
Blindness and vision loss (aggregate)	Prevalence	1995–2023	−0.46	−0.53, −0.40
Blindness and vision loss (aggregate)	YLD	1990–2019	−1.75	−1.80, −1.69
Blindness and vision loss (aggregate)	YLD	2019–2023	+0.28	−0.50, 1.06
Blindness	Prevalence	1990–2019	−2.77	−2.80, −2.73
Blindness	Prevalence	2019–2023	+0.29	−0.23, 0.81
Blindness	YLD	1990–2019	−2.73	−2.76, −2.70
Blindness	YLD	2019–2023	+0.26	−0.25, 0.76
Severe vision loss	Prevalence	1990–1998	−1.69	−1.80, −1.58
Severe vision loss	Prevalence	1998–2023	−1.22	−1.24, −1.20
Moderate vision loss	Prevalence	1990–2008	+1.01	0.69, 1.33
Moderate vision loss	Prevalence	2008–2023	+0.09	−0.28, 0.47
Moderate vision loss	YLD	1990–2008	+1.04	0.75, 1.34
Moderate vision loss	YLD	2008–2023	+0.11	−0.30, 0.52

Note. Segment CIs are from OLS on the shorter segment (as few as five annual points for the most recent post-breakpoint segments) and are not further HAC- or Monte-Carlo-adjusted; they are reported as a sensitivity check on segment direction, not as precision estimates on a par with Table 1. EAPC = estimated annual percentage change; YLD = years lived with disability; CI = confidence interval; OLS = ordinary least squares; HAC = heteroskedasticity- and autocorrelation-consistent. Source: output/segment_specific_eapc.csv.

**Table 5:** Composition and Trend of Five Tracked, Non-Overlapping Causes of Vision Impairment, Both Sexes, Saudi Arabia, 1990–2023, With Three Confidence-Interval Methods.

Cause	Prevalence Share, 1990 (%)	Prevalence Share, 2023 (%)	Point Change (pp)	Crude Prevalence EAPC	HAC-Robust 95% CI	Robustness Verdict
Uncorrected refractive error	62.2	69.4	+7.1	+0.88	−0.92, 2.72	Suggestive only
Cataract	25.5	18.5	−7.0	−0.76	−1.59, 0.08	Suggestive only
Diabetes mellitus (type 1 + type 2)	1.8	7.0	+5.3	+4.47	3.30, 5.65	Confirmed increasing
Glaucoma	8.1	3.2	−4.9	−2.99	−3.79, −2.17	Confirmed decreasing
Age-related macular degeneration	2.4	1.9	−0.5	−0.72	−3.34, 1.97	Suggestive only

Note. Shares are each cause’s Number as a percentage of the summed Number of the five tracked causes (not of the full “blindness and vision loss” envelope; see Section 7.2 and Section 8.6). “Diabetes and kidney diseases” is omitted as numerically identical to “diabetes mellitus” in this extract. “Diabetes mellitus” is GBD’s cause label; the underlying ocular pathology is diabetic retinopathy (see Section 6.3). Within diabetes mellitus, the type 2 sub-component rose faster (crude prevalence EAPC = +4.49%/year, confirmed) than type 1 (+2.43%/year, confirmed). “Confirmed”/”suggestive” verdicts as in Table 1; the age-standardised version of this table was not calculable, since an age-standardised cause-specific rate extract was not obtained (see Section 6.4). EAPC = estimated annual percentage change; HAC = Newey-West heteroskedasticity- and autocorrelation-consistent; CI = confidence interval; pp = percentage points; GBD = Global Burden of Disease. Source: output/cause_proportion_change.csv, output/cause_eapc_crude_COMBINED.csv, output/envelope_coverage_check.csv.

**Table 6:** Severity Distribution of Diabetes-Attributable Vision Impairment, Both Sexes, Saudi Arabia, 1990 and 2023.

Severity Category	Prevalence Share, 1990 (%)	Prevalence Share, 2023 (%)	YLD Share, 1990 (%)	YLD Share, 2023 (%)
Moderate vision loss	56.3	63.5	17.8	22.4
Blindness	26.8	27.7	50.6	58.9
Severe vision loss	16.9	8.9	31.5	18.7

Note. Shares sum to 100% within each measure and year (diabetes returned no rows for presbyopia, which GBD attributes solely to uncorrected refractive error). YLD = years lived with disability; GBD = Global Burden of Disease. Source: output/diabetes_severity_distribution.csv.

## Data Availability

The data that support the findings of this study are available in the Global Burden of Disease (GBD) 2023 Results Tool, Institute for Health Metrics and Evaluation (IHME), at https://vizhub.healthdata.org/gbd-results/ (accessed on 31 July 2026). The raw data extracts, replication scripts, and analysis output used in this study have been deposited in the Open Science Framework (OSF) and are publicly available at https://osf.io/dn5f6/overview?view_only=be33e1f620c346cc80c19e0d3abc3137 (accessed on 31 July 2026). Additional analysis scripts (segment-specific EAPC, sex-difference interaction test, Durbin-Watson autocorrelation check, and Monte Carlo uncertainty propagation) have been added to the same OSF repository.
